# Health – Nutrition - Fitness - Wellness- an actual challenge

**Published:** 2012-12-25

**Authors:** F Popa

**Affiliations:** Carol Davila University of Medicine and PharmacyRomania

The second edition of the ** International Congress Health-Nutrition-Fitness-Wellbeing - SANABUNAINT 2012 ** took place in Falticeni, during 19-21 of October 2012. The event, which was organized under the patronage of the Romanian Patriarchy, the Romanian Academy, the Ministry of Public Health, the Ministry of Agriculture and Rural Development, the Ministry of Education, Research, Youth and Sport, has benefited from the presence of some important personalities. Lots of physicians took part in the event, many of whom being youngsters or even students.

**Fig. 1:**
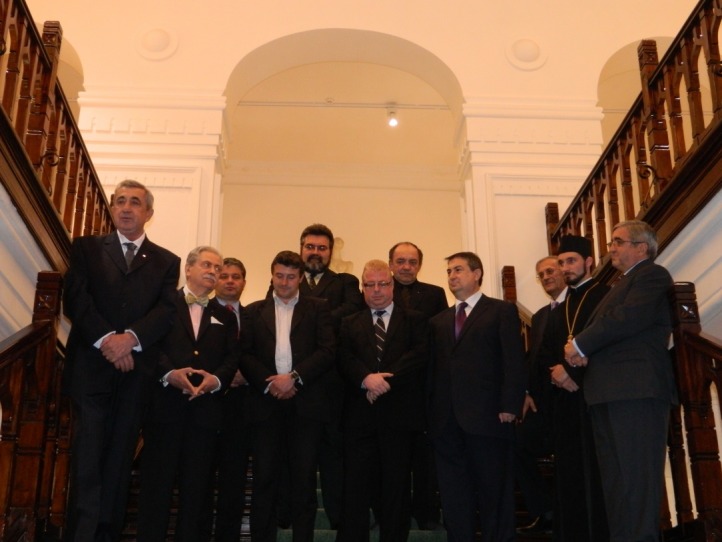
“Ion Irimescu” Art Museum - The Opening Ceremony of the Congress

The event, which took place in the context of the 9th edition of the **Apple Festival - “The Apples of Radaseni”**, and as an expression of the responsible preoccupation of the **Falticeni Town Hall** and **Suceava Town Council** in promoting the Public-Civic-Private Partnership, while considering the entire series of potential benefits (health, social, direct and indirect economical, environmental), under the slogan **“Pilot Station” Falticeni and an assumed challenge: Constructing a Central Regional and Eastern-European Model of Thinking and Acting”**, was held for two days and has brought into light discussion themes such as the following: The Danube Delta - TD Education Research Innovation Development; Communication and Information Technology: Protecting, Promoting Health and Preventing Diseases – Re; Health-Nutrition-Fitness-Wellness - an actual challenge; etc. 

**Fig. 2:**
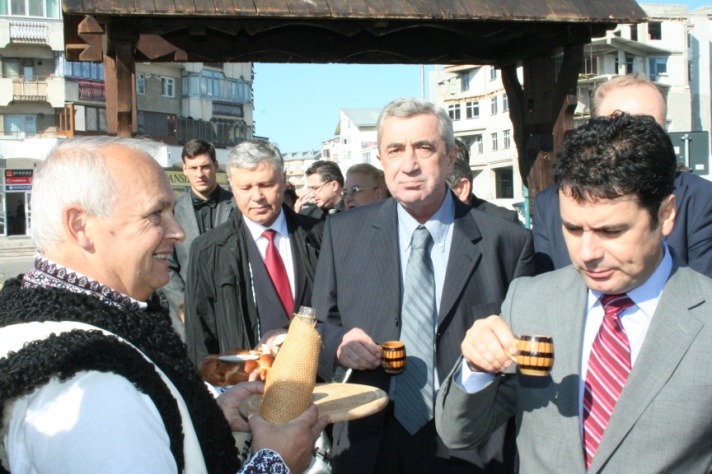
The visit of the participants to the “Apple Festival” in the central market of Falticeni town

**Fig. 3:**
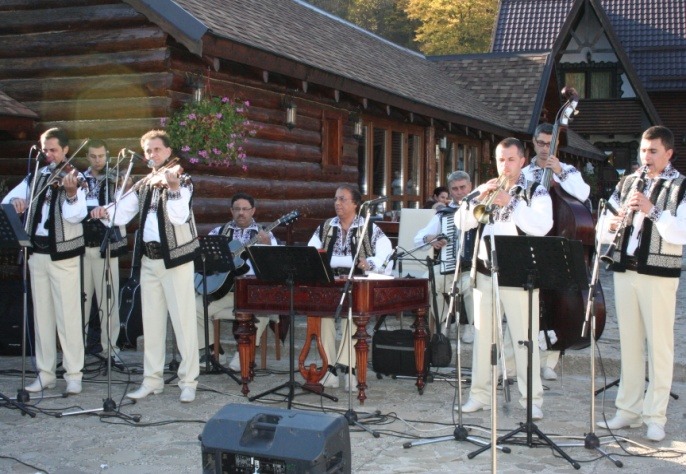
Folkloric Show offered in the honor of the participants by “Ciprian Porumbescu” Folkloric Band

A section of round tables and a rich and useful explosion of medical books, belonging to the excellent and famous “Carol Davila” University Press, have completed the program. The works of the Congress were closed with the awards ceremony for the best papers and a magnificent show of “Ciprian Porumbescu” folkloric group.

